# Injectable and Antioxidative HT/QGA Hydrogel for Potential Application in Wound Healing

**DOI:** 10.3390/gels7040204

**Published:** 2021-11-09

**Authors:** Yikun Ren, Dan Zhang, Yuanmeng He, Rong Chang, Shen Guo, Shanshan Ma, Minghao Yao, Fangxia Guan

**Affiliations:** School of Life Science, Zhengzhou University, 100 Science Road, Zhengzhou 450001, China; renyikun1996@126.com (Y.R.); z13132572181@163.com (D.Z.); h1914375052@163.com (Y.H.); a15738343526@163.com (R.C.); 15137690960@163.com (S.G.); mashanshan84@163.com (S.M.)

**Keywords:** injectable hydrogel, antioxidant, biocompatibility, wound healing

## Abstract

Hydrogels have gained a niche in the market as wound dressings due to their high water content and plasticity. However, traditional hydrogel wound dressings are difficult to fully adapt to irregular-shaped wound areas. Additionally, excessive reactive oxygen species (ROS) accumulated in the damaged area impede the wound healing process. Therefore, hydrogels with injectable and antioxidant properties offer promising qualities for wound healing, but their design and development remain challenges. In this study, HT/QGA (tyramine-grafted hyaluronic acid/gallic acid-grafted quaternized chitosan) hydrogels with injectable and antioxidant properties were prepared and characterized. This hydrogel exhibited excellent injectability, favorable antioxidant activity, and good biocompatibility. Moreover, we evaluated the therapeutic effect of HT/QGA hydrogel in a full-thickness skin injury model. These results suggested that HT/QGA hydrogel may offer a great potential application in wound healing.

## 1. Introduction

Skin, as the largest human organ, is the body’s first line of defense [1,2]. Wound is a defect in or a breakage of the skin caused by physical, chemical or thermal damage. They can be classified based on the degree of layers and area of skin affected, including superficial wounds, partial thickness wounds, and full thickness wounds [3]. Wound healing is the result of a series of biological molecules and stem cells working together to promote wound repair at all levels, which is considered as one of the most complex dynamic biological processes. This complex process can be divided into four parts: hemostasis, inflammation, proliferation, and remodeling [4,5,6]. An ideal wound dressing should fulfill requirements such as maintaining a moist environment, enhancing epidermal migration, promoting angiogenesis, and providing protection against bacterial infection [7].

Hydrogels are defined as three-dimensional cross-linked polymeric network structures with the ability to hold a large amount of water in their networks and retain their structures after swelling [8]. Traditional wound dressing products, such as gauze and bandages, are inexpensive and can offer some protection. However, they tend to adhere to the wound sites, causing secondary injury during dressing changes once the absorbed exudate dries out [9]. Therefore, hydrogel is considered to be an ideal dressing candidate, because of its 3D structure, good permeability, excellent biocompatibility, and its ability to provide a wet environment for wound repair, which overcomes the shortcomings of traditional dressings [4,10].

Hyaluronic acid (HA) is a natural macromolecule polysaccharide composed of N-acetylamino-glucose and D-glucuronic acid, and it is an important component of extracellular matrix [11]. HA-Tyramine (HT) is a phenolic hydroxyl-riched polymer synthesized by grafting tyramine with hyaluronic acid. HT has already been developed as a protein drug delivery system [12,13], and is widely used in tissue engineering [14,15]. It offers the advantages of cell compatibility, biodegradability, and non-immunogenicity [16]. Chitosan (CS) is a natural polysaccharide prepared by the deacetylation of chitin [17,18]. It has drawn increasing attention for its biomedical applications, owing to its biocompatibility, biodegradability, non-toxicity, and low-immunogenicity [19]. However, the application of CS is limited due to its insoluble property in water. Compared with chitosan, quaternized chitosan (QCS) has been widely used due to its excellent antibacterial activity and improved water solubility. Gallic acid (GA) is an antioxidant polyphenol that features neuroprotective functions in different models of neurodegeneration, neurotoxicity, and oxidative stress [20]. Gallic acid has been conjugated onto QCS chains to obtain antioxidant QGA conjugate. Finally, HT and QGA conjugate was enzymatically crosslinked via a reaction catalyzed by horseradish peroxidase (HRP) using hydrogen peroxide (H_2_O_2_) as substrate.

Reactive oxygen species (ROS) are important intermediate products in human metabolism and play a role in maintaining the normal physiological activities of human beings [21]. Although low levels of reactive oxygen species (ROS) are conducive to normal wound healing by stimulating cell migration and angiogenesis, the overexpression of ROS can cause a series of inflammatory reactions, and hinder or even endanger wound healing [22,23]. Antioxidant hydrogel is considered as one of the important biomaterials for ROS removal due to their outstanding biological compatibility and antioxidant property.

Historically, hydrogels were pre-formed, and the delivery of these materials to target sites in patients necessitated the use of highly invasive surgical procedures [24]. However, influential work in the late 1990s demonstrated that hydrogel precursors could be injected through a standard syringe and crosslinked locally [25]. Injectable hydrogels provide certain advantages, such as filling wound sites with irregular space, adhering to wounds, and the ability to perform the in situ encapsulation of bio-active molecules and cells that are important for enhanced skin tissue regeneration [9].

In this study, HT/QGA hydrogel was prepared and characterized. This hydrogel exhibited good injectability, antioxidant activity, stability and biocompatibility. Furthermore, it demonstrated good therapeutic effects in full-thickness skin injury model. These results indicated that HT/QGA hydrogel may offer great potential for application in wound dressing.

## 2. Results and Discussion

### 2.1. Synthesis and Characterization of HT and QGA

**Figure 1:**
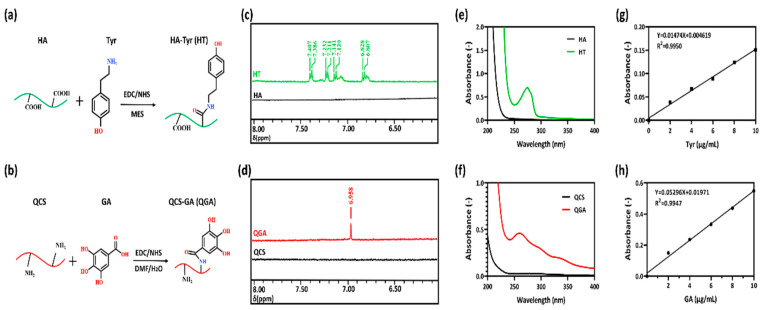
Synthetic scheme of (**a**) hyaluronic acid-tyramine (HT) and (**b**) quaternized chitosan-gallic acid (QGA) conjugate; (**c**) ^1^H NMR spectra of HA and HT conjugate; (**d**) ^1^H NMR spectra of QCS and QGA conjugate; (**e**) the UV-vis spectra of HA and HT; (**f**) the UV-vis spectra of QCS and QGA; (**g**) the standard curve of tyramine; (**h**) the standard curve of GA.

The reaction of hyaluronic acid with Tyr (tyramine) occurred between the carboxylic group of hyaluronic acid and the amino group of Tyr to form the amide linkage, using the EDC/NHS-mediated coupling reaction. The reaction of quaternized chitosan with GA occurred between the amino group of quaternized chitosan and the carboxylic group of GA to form the amide linkage, also using the EDC/NHS-mediated coupling reaction. The synthesis scheme of HT and QGA polymers is shown in Figure 1a,b.

In this study, we confirmed the structure of HT and QGA polymers via ^1^H NMR spectra. As shown in Figure 1c,d and Figure S1, the presence of Tyr in HT was verified by the resonance peaks at 7.12–7.40 ppm and 6.82 ppm, which represent the benzene ring in Tyr structure [11]. The resonance peak of GA was at 6.95 ppm, which represents the benzene ring in GA structure [26].

The grafting ratio of Tyr was calculated by measuring tyramine absorbance at 275 nm of 1 mg/mL HT solution via UV-vis spectra (Figure 1e). The measured absorbance was compared to a tyramine standard curve composed of 2, 4, 6, 8, and 10 µg/mL of tyramine in ultrapure water (Figure 1g). The grafting ratio of Tyr in HT was 4.72%; in another word, there were 340 μmol phenol groups in 1 g of HT.

The grafting ratio of GA was calculated by measuring GA absorbance at 258 nm of 10 mg/mL QGA solution via UV-vis spectra (Figure 1f). The absorbance measured was compared to a GA standard curve composed of 2, 4, 6, 8, and 10 µg/mL of GA in ultrapure water (Figure 1h). The grafting ratio of GA in QGA was 8.31%; in another word, there were 488.48 μmol phenol groups in 1 g of QGA.

### 2.2. Hydrogel Fabrication, Gelation Time, Injectability and Water Content

**Figure 2:**
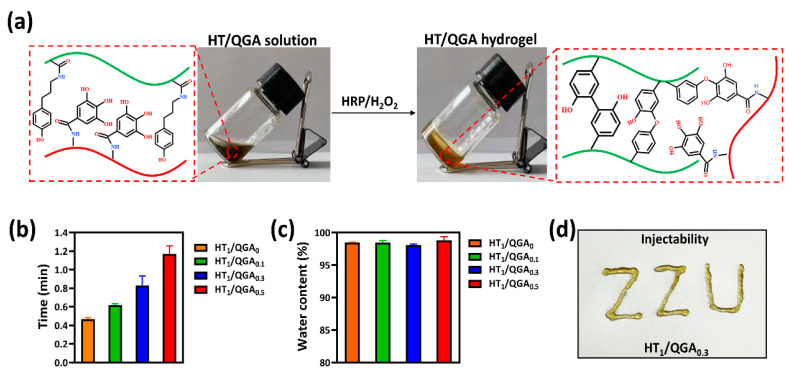
(**a**) Scheme of the HT/QGA hydrogel cross-linked by HRP and H_2_O_2_. (**b**) Gelation time, (**c**) water content and (**d**) injectability of HT/QGA hydrogel. Mean ± SD, *n* = 3.

The HT/QGA hydrogels, including HT_1_/QGA_0_, HT_1_/QGA_0.1_, HT_1_/QGA_0.3_, and HT_1_/QGA_0.5_, were fabricatedby using the HRP/H_2_O_2_ catalytic method. The fabrication and crosslinking mechanism of HT/QGA hydrogels are shown in Figure 2a. In this system, crosslinked HT act as the main backbone. The cross-linking of the phenol moieties of HT conjugates takes place through either C–C bonds between the ortho- carbons of the aromatic ring or through the C–O bonds between the ortho-carbons and the phenolic oxygen in the presence of HRP and H_2_O_2_. GA as a powerful antioxidant composition, also competitively consumes H_2_O_2_, so that the crosslinking speed and density of hydrogel decreases with the growth of the QGA proportion. The gelation time of the hydrogels are shown in Figure 2b. With the increase of QGA concentration, the gelation time of HT/QGA hydrogel is longer. It indicates that the higher the concentration of QGA, the longer the hydrogel formation. In total, all the gelation time of HT/QGA hydrogel are under two minutes, which makes it a promising material for clinical usage. As shown in Figure 2c, the water contents of hydrogels are all higher than 98%, which is close to the natural extracellular matrix. As shown in Figure 2d, the HT_1_/QGA_0.3_ hydrogel was injectable. This gave it the ability to completely adapt to the shape of wound.

### 2.3. Rheological and Morphological Properties of Hydrogel

**Figure 3:**
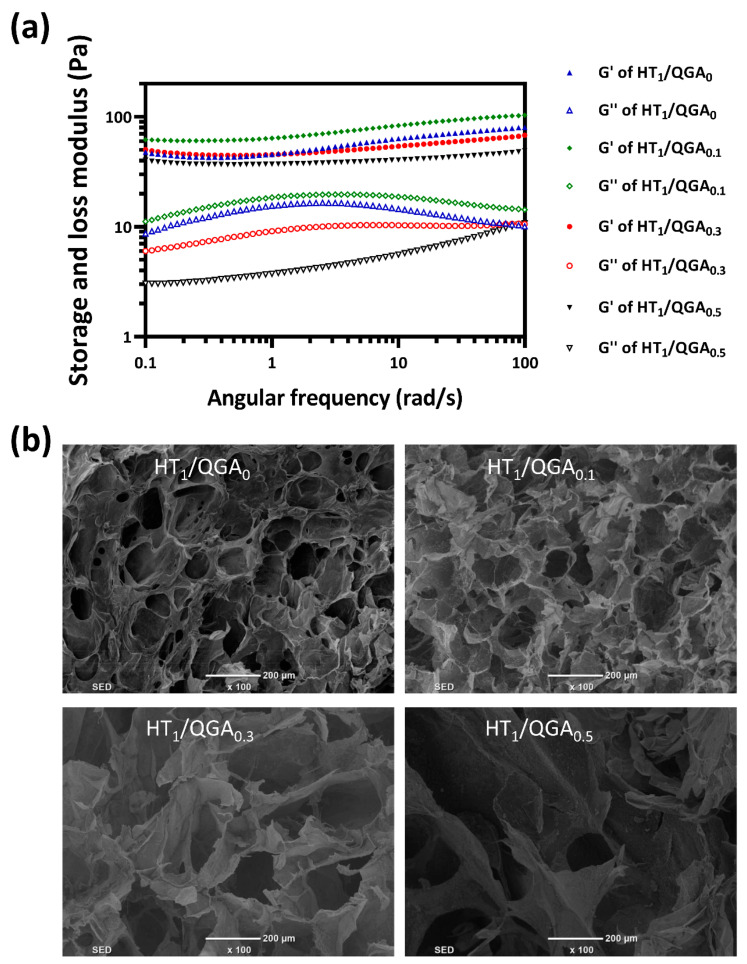
(**a**) The rheological properties of HT/QGA hydrogel; (**b**) the morphology of HT/QGA measured by SEM.

The mechanical strength of HT/QGA hydrogel was further evaluated by rheological experiments (Figure 3a). The results indicated that the storage modulus (G’) are larger than loss modulus (G”) of HT/QGA hydrogel at all tested frequencies (0.1 to 100 rad/s), which confirms their gel-like behavior. Moreover, the G’ and G” of HT/QGA hydrogels remained constant throughout the tested frequency, indicating that these hydrogels offer good mechanical stability [27]. As shown in Figure 3b, the morphology of HT/QGA hydrogels was observed with a scanning electron microscope (SEM). All hydrogels displayed a relatively uniform porous structure, and the pore diameter ranged from dozens to hundreds of microns. Moreover, the incorporation of QGA enlarged the pore diameter. A higher content of QGA leads to a larger pore size. The porous structure of the hydrogels is beneficial to the absorption of wound excretion.

### 2.4. In Vitro Stability and Biodegradability Tests of Hydrogel

**Figure 4:**
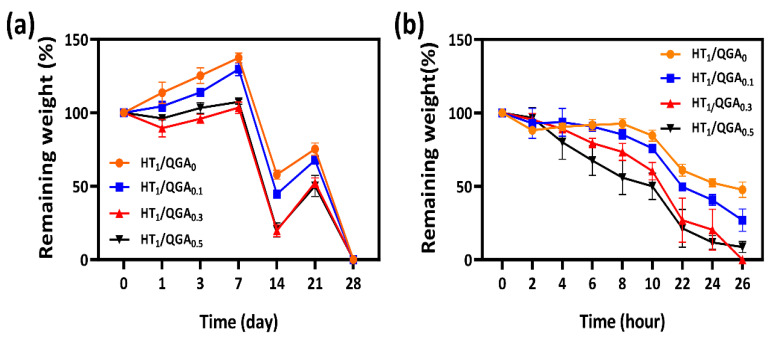
(**a**) In vitro stability and (**b**) biodegradability of HT/QGA hydrogels. Mean ± SD, *n* = 3.

The in vitro stability of hydrogels was measured by immersing the hydrogels in PBS and observing the number of days required for their degradation. As shown in Figure 4a, the degradation rate of HT_1_/QGA_0_ was the slowest. With the increase of QGA concentration, the degradation rate of HT/QGA hydrogels was faster, proving that the higher the concentration of QGA, the lower the crosslinking density and stability of the hydrogel formation. In addition, the biodegradability of the hydrogels was also measured by immersing them in PBS containing 20 Units/mL hyaluronidase and observing the number of days they took to degrade. As shown in Figure 4b, the HT_1_/QGA_0_ hydrogel demonstrated the slowest enzymatic hydrolysis rate, and the enzymatic hydrolysis rate of HT/QGA hydrogels increased with the increase of QGA concentration. The results showed that the stability and biodegradability in vitro could be controlled by altering the mass of QGA.

### 2.5. Antioxidant Efficiency of Hydrogel

**Figure 5:**
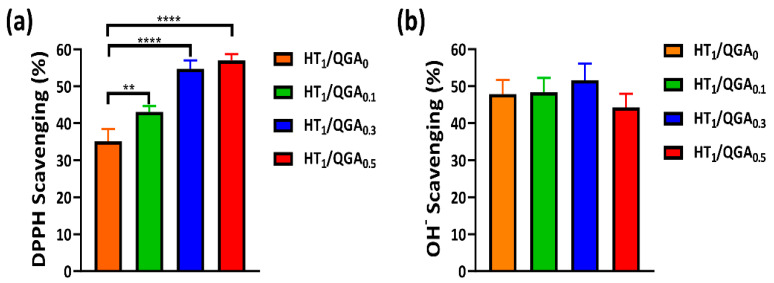
Antioxidant efficiency of HT/QGA hydrogel. (**a**) DPPH radicals’ and (**b**) hydroxyl radicals’ scavenging ability in hydrogel. ** *p* < 0.01, **** *p* < 0.0001, mean ± SD, *n* = 3.

The introduction of GA into the hydrogels was due to its good antioxidant activity. We have evaluated the antioxidant activity of the hydrogels by testing the DPPH (nitrogen radicals) and hydroxyl radicals’ scavenging rates. As shown in Figure 5a, with the increase of QGA concentration, the DPPH scavenging rate gradually increased. Compared to the HT_1_/QGA_0_ hydrogel, DPPH scavenging rate of HT_1_/QGA_0.1_, HT_1_/QGA_0.3_, and HT_1_/QGA_0.5_ hydrogels increased significantly from 35.1% to 43.1%, 54.7%, and 57.0%, respectively. This indicates that QGA increased the DPPH’s scavenging rate due to the phenolic hydroxyl group in QGA. As shown in Figure 5b, the OH^−^ scavenging rate of HT_1_/QGA_0_ hydrogel reached to 48.0%, and the scavenging rates of HT_1_/QGA_0.1_, HT_1_/QGA_0.3_, and HT_1_/QGA_0.5_ hydrogels were 48.4%, 54.7%, and 44.3%, respectively. There is no statistically significance between these hydrogels, indicating that QGA has no positive influence on the OH^−^ scavenging property.

### 2.6. Cytocompatibility and Hemolysis of Hydrogel

**Figure 6:**
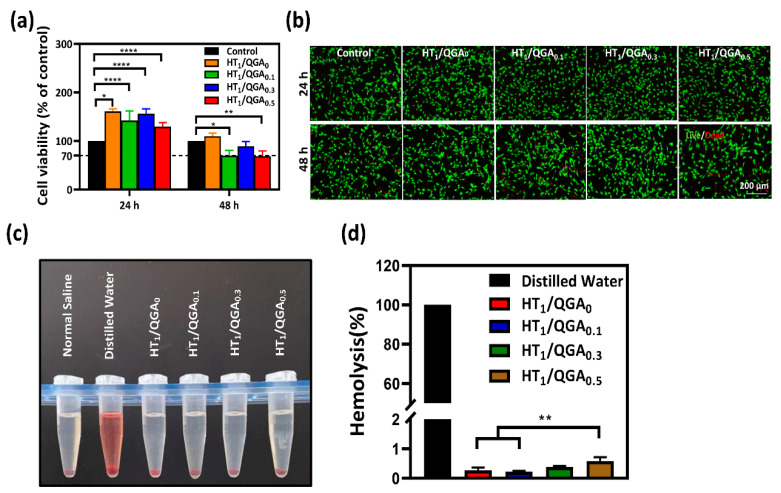
(**a**) BMSCs viability of HT/QGA hydrogel extracts by CCK-8; (**b**) live (Calcein-AM)/dead (PI) dyeing of BMSCs; (**c**) photograph of hemolysis test and (**d**) hemolysis ratio of HT/QGA hydrogels. * *p* < 0.05, ** *p* < 0.01, **** *p* < 0.0001, mean ± SD, *n* = 3.

The basic property required for biomaterials is good compatibility [28,29]. According to the assessment criteria of the International Organization for Standardization (ISO) 10993-5, hydrogels are safe and can be applied to clinical use under the condition ofa cell viability rate higher than 70%. Therefore, a cytocompatibility test (CCK-8 assay) with BMSCs was performed to evaluate the hydrogels’ in vitro cytotoxicity. On the first day, the cell viability rates of HT_1_/QGA_0.1_, HT_1_/QGA_0.3_, and HT_1_/QGA_0.5_ hydrogels increased to 161.2 ± 5.3%, 142.1 ± 20.1%, 156.5 ± 9.8%, and 129.7 ± 8.1%, respectively; All these values were significantly higher than those of the control group. This indicates that the hydrogels were more than 100% compatible with the BMSCs and promoted cell proliferation. On the second day, the cell viability rates of the HT_1_/QGA_0_, HT_1_/QGA_0.1_, HT_1_/QGA_0.3_, and HT_1_/QGA_0.5_ hydrogels were 109.8 ± 6.2%, 69.7 ± 10.8% (significantly lower than the control group, *p* < 0.05), 89.0 ± 10.2%, and 68.6 ± 10.8% (significantly lower than the control group, *p* < 0.01), respectively. Apart from the HT_1_/QGA_0_, the HT_1_/QGA_0.3_ hydrogel showed the highest cytocompatibility compared with the other groups (Figure 6a). Calcein-AM/PI assay was also used to evaluate cell viability after 24 and 48 h of contact with the hydrogel extraction. As shown in Figure 6b, the cell viability staining was consistent with the CCK-8 result. The BMSCs in all the groups were still stained with a bright green color, indicating that HT/QGA hydrogels exert no obvious toxicity on cells.

The hemolysis ratio is the most simple and effective method to measure the blood compatibility of hydrogels. According to the assessment criteria of the International Organization for Standardization (ISO) 10993-4, hydrogels are safe and can be applied to clinical use under the condition of a hemolysis ratio lower than 5% [30]. As shown in Figure 6c,d, the extraction of hydrogels was as clear as that of the normal saline group, and the hemolysis ratio of the HT_1_/QGA_0_, HT_1_/QGA_0.1_, HT_1_/QGA_0.3_, and HT_1_/QGA_0.5_ hydrogels were 0.27 ± 0.09%, 0.22 ± 0.03%, 0.39 ± 0.04%, and 0.58 ± 0.14%, respectively. The hemolysis ratio of the HT/QGA hydrogels gradually increased with the growing QGA content, but all the hydrogel groups displayed a hemolysis ratio far lower than 5%. These results indicate that HT/QGA hydrogels have excellent blood compatibility as wound dressings.

### 2.7. Biocompatibility and Biodegradability of Hydrogel In Vivo

**Figure 7:**
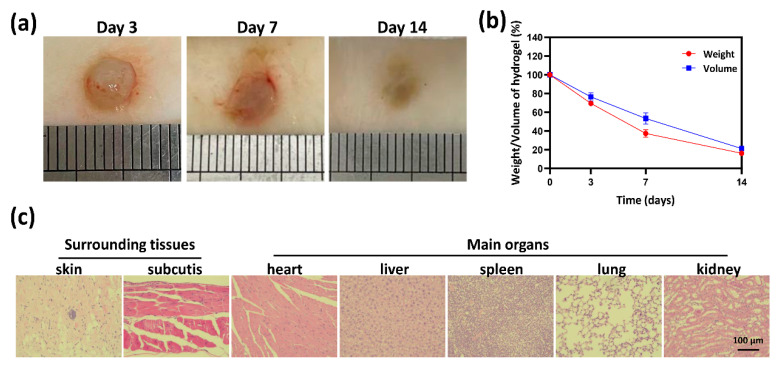
Morphology (**a**) and degradation rate (**b**) of hydrogels in Kunming mice. (**c**) HE staining of surrounding tissues and main organs (heart, liver, spleen, lung, and kidney) on the fourteenth day. Mean ± SD, *n* = 3.

Based on the above experimental data, especially the antioxidative activity and cytocompatibility, the HT_1_/QGA_0.3_ hydrogel was selected for further investigation in vivo. It’s in vivo biocompatibility and biodegradability were evaluated by subcutaneous injection of hydrogels in mice. The mass and volume of the subcutaneous hydrogels were measured on the third, seventh, and fourteenth days after injection, representing the biodegradability of the hydrogels in vivo. As shown in Figure 7a,b, there was no obvious pathological change observed on the macro level, and all the hydrogels degraded gradually. Tissues near the subcutaneous injection site and main organs were taken for HE staining, as shown in Figure 7c. Barely inflammatory cells were observed in skin, subcutaneous tissue, heart, liver, spleen, lung, and kidney. These results indicate that HT/QGA hydrogel demonstrates good biocompatibility and biodegradability in vivo.

### 2.8. In Vivo Wound Healing in a Full-Thickness Skin Defect Model

**Figure 8:**
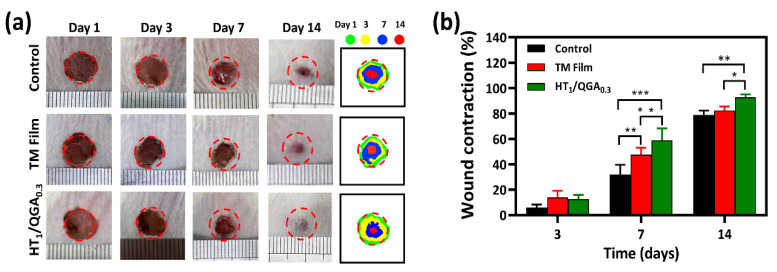
(**a**) Photographs and schematic diagram of wound area on 1st, 3rd, 7th, and 14th day of Control, TM film and HT_1_/QGA_0.3_ hydrogel group; (**b**) Wound contraction of each group. * *p* < 0.05, ** *p* < 0.01, *** *p* < 0.001, mean ± SD, *n* = 3.

Photographs of the wound area were taken on the third, seventh, and fourteenth day after treatment (Figure 8a). On the third day, wound healing was better in the HT_1_/QGA_0.3_ group than in the Control and TM Film groups. On the seventh day, the wound area in the HT_1_/QGA_0.3_ group was the smallest, proving that the wound healing rate was faster in the HT_1_/QGA_0.3_ group. On the fourteenth day, the HT_1_/QGA_0.3_ group still showed a higher rate of healing. Compared with the Control group, the HT_1_/QGA_0.3_ group showed a significantly enhanced wound repair rate on the seventh and fourteenth day (Figure 8b). These results suggested that HT_1_/QGA_0.3_ hydrogel promotes wound healing.

## 3. Conclusions

In this study, a series of injectable and antioxidative HT/QGA hydrogels based on hyaluronic acid-graft-tyramine (HT) and quaternized chitosan-graft-gallic acid (QGA) were successfully prepared through the crosslinking of catechol groups with HRP/H_2_O_2_ as the initiator system. These hydrogels provide good stability, antioxidant properties, cytocompatibility, hemocompatibility, histocompatibility, and biodegradability. Importantly, HT_1_/QGA_0.3_ hydrogel demonstrated a faster rate of wound closure than the Control and TM Film groups, suggesting that HT_1_/QGA_0.3_ hydrogel can significantly promote wound healing. These results demonstrated that HT/QGA hydrogels offer great potential in clinical applications.

## 4. Materials and Methods

### 4.1. Materials

The HA sodium salt (from *Streptococcus equi*), tyramine (Tyr), Gallic acid (GA), 1-ethyl-3-(3-dimethylaminopropyl)-carbodiimide (EDC), and N-hydroxy succinimide (NHS) were purchased from Shanghai Aladdin Biochemical Technology Co., Ltd. (Shanghai, China). The 2-(N-Morpholino) ethanesulfonic acid (MES), Hyaluronidase (enzymatic activity: 608 units mg^−1^), Safranin O, and dialysis membrane (molecular cut off = 3500 Da) were bought from Beijing Solarbio Technology Co., Ltd. (Beijing, China). The quaternized chitosan (QCS) was purchased from Shanghai Yuanye Biotechnology Co., Ltd. (Shanghai, China). The N,N-dimethylformamide (DMF) was bought from Tianjin Yongda Chemical Reagent Co., Ltd. (Tianjin, China). The mouse bone mesenchymal stem cells (BMSC) were obtained from the Cell Bank, Type Culture Collection, Chinese Academy of Sciences, Shanghai, China. The CCK-8 was obtained from Invigentech (Irvine, CA, USA). The chloral hydrate was purchased from Shanghai Yien Chemical Technology Co., Ltd. (Shanghai, China). The Tegaderm film was obtained from 3M Health Care (Saint Paul, MN, USA). The Kunming mice were obtained from the Experimental Animal Center of Zhengzhou University.

### 4.2. Synthesis and Characterization of HT and QGA

Tyramine was grafted onto the HA chain to obtain HT conjugate according to our previous method [16]. Briefly, the HA sodium salt was dissolved in 100 mL of MES solution. EDC and NHS were then added to activate the carboxylic group of HA. After the activation of HA, tyramine was added and reacted at room temperature for 24 h. Gallic acid was grafted onto the QCS chain to obtain QGA polymer according to the previous procedure with some modifications [31]. Briefly, the carboxylic group of GA was first activated with EDC and NHS in a co-solvent of water and DMF (volume ratio of 3:2) for 1 h. The activated GA solution was then added to the dissolved QCS solution and reacted at room temperature for 24 h. The mixed solution was then transferred to a dialysis bag and dialyzed against distilled water for 3 days. Next, the solution was freeze-dried to obtain HT and QGA polymers. Then, HT and QGA were characterized by 600 MHz ^1^H NMR spectroscopy (AS400, OXFORD instruments, Abingdon, UK). The Tyr content of HT and GA content of QGA were measured quantitatively at 275 nm and 258 nm using a UV–vis spectrophotometer (V-750 UV/vis/NIR, Jasco, Tokyo, Japan).

### 4.3. Hydrogel Fabrication, Injectability, Gelation Time and Water Content

First, 2.0% HT and 2.0% QGA solutions (*w*/*v*) were prepared. The two solution were then mixed by different proportions to form 1%HT/0%QGA, 1%HT/0.1%QGA, 1%HT/0.3%QGA, and 1%HT/0.5%QGA mixtures. The resulting mixtures were denoted as HT_1_/QGA_0_, HT_1_/QGA_0.1_, HT_1_/QGA_0.3_, and HT_1_/QGA_0.5_, respectively. When the percentage of QGA was larger than 0.7%, HT/QGA solution could not form a homogeneous and stable hydrogel. Finally, the mixtures were transferred to clean petri dishes and crosslinked by 1 U/mL HRP and 0.01% H_2_O_2_. The gelation time was determined by tube inversion method.

The injectability of the hydrogels was measured by a 1 mL syringe. The water content of the hydrogels was measured according to the following steps. 100 µL of hydrogels were soaked in PBS at 37 °C for 24 h. The water on the surface of the hydrogels wasdried out and weighed, namely, W_0_. The hydrogels were freeze-dried and weighed, namely, W_1_. The water content of the hydrogels was calculated by using this equation:

Water content of hydrogel (%) = (W_0_ − W_1_)/W_0_ × 100

### 4.4. Morphology and Rheological Properties of Hydrogel

The morphology of the hydrogels were examined by using a scanning electron microscope (SEM) (FEI Quanta200, Netherlands). The hydrogels were prepared and frozen in a −80 °C refrigerator, and freeze-dried in a freeze-vacuum dryer. After immersing the dried hydrogels in liquid nitrogen for 30 s, a sharp blade was used to cut out a flat cross section. Then, a vacuum coating apparatus was used to spray gold on the section. Next, the hydrogels were observed under the scanning electron microscope.

The rheological behavior of the HT/QGA hydrogels was evaluated by detecting the storage modulus and loss modulus of the hydrogels using a rheometer platform (Leica DHR2, Germany). The dynamic oscillation scanning frequency ranged from 0.1 to 100 rad/s; the temperature and strain were set as 37 °C and 1%, respectively [32].

### 4.5. In Vitro Stability and Biodegradability Test of Hydrogel

The stability of the hydrogels was investigated by incubating them in a PBS solution at 37 °C. The biodegradability of the hydrogels was investigated by incubating the hydrogels in a PBS solution containing hyaluronidase. For the stability and biodegradability test, 100 µL hydrogels were prepared in a 2.0 mL centrifuge tube and weighed (W_i_). Next, the hydrogels were incubated in 1 mL of PBS solution with/without 20 Units/mL hyaluronidase at 37 °C. At intervals, the media were removed, and the weights of the remaining hydrogels were measured (W_d_) before the fresh media were added. The percentage of the remaining hydrogels was calculated by using this equation:

Weight of remaining hydrogel (%) = W_d_/W_i_ × 100

### 4.6. Antioxidant Efficiency of Hydrogel

The antioxidant efficiency of the hydrogels was tested by measuring their capacity to scavenge 1,1-diphenyl-2-picrylhydrazyl (DPPH) (nitrogen radicals) and hydroxyl radicals.

The scavenging rate of the DPPH radicals was evaluated through the following procedure. Briefly, 300 µL of hydrogels were prepared and immersed in 1 mL of ethanol for 0.5 to 1 h. Next, 100 µL of 0.5 mM DPPH ethanol solution was added and incubated in the dark for 1 h. The control group used 300 µL of distilled water instead of hydrogel. Finally, the absorbance of ethanol solution at 517 nm was measured. The experiment was repeated three times.

The scavenging rate of the OH^−^ radicals was measured according to the following steps: 300 µL of hydrogels were prepared and immersed in the mixed solution of 2 mM FeSO_4_ and 1 mM Safranin O for 10 min. For the blank and control groups, 300 µL distilled water was used to replace hydrogels. Next, 6% H_2_O_2_ was added and incubated at 50 °C for 1 h. For the control group, distilled water was used instead of H_2_O_2_. Finally, the absorbance of the solution at 492 nm was measured. The experiment was repeated three times.

### 4.7. Cytocompatibility and Hemolysis of Hydrogel

The hydrogels were added to 1 mL of cell culture media respectively and incubated at 37 °C for 24 h to form hydrogel extraction. The BMSCs were seeded in a 96 well plate at a density of 3000 cells per well. After being cultured for 24 h, the media was discarded and replaced with hydrogel extraction. After 24 and 48 h, the cell viability was evaluated by CCK-8 and Calcein-AM/PI (Live/Dead) Kit assay.

The hemolysis ratio of the hydrogels was tested according to the following steps. Fresh mouse heart blood was collected and placed into an anticoagulant tube for temporary storage. The HT/QGA hydrogels were immersed in normal saline and incubated at 37 °C for 24 h. Next, 20 µL of blood was added and incubated for 3 h at 37 °C. At the same time, water was set as the positive control and normal saline was set as the negative control. After 3 h, the mixed solutions were centrifuged at 2000 rpm for 5 min. After photographing, the absorbance of the supernatant at 545 nm was measured. The experiment was repeated three times.

### 4.8. Biocompatibility and Biodegradation of HT Hydrogel In Vivo

The HT/QGA pre-hydrogel solution was prepared under aseptic conditions. The mice were anesthetized and shaved. The HRP/H_2_O_2_ was added before injection, then the solution was injected and gelled subcutaneously in situ. On days 3, 7, and 14 after injection, the mice were anesthetized and perfused. The hydrogels were photographed, the weight and volume of the hydrogels were recorded. The tissues around the subcutaneous injection site and main organs (heart, liver, spleen, lung, and kidney) were taken for hematoxylin-eosin (H&E) staining to observe whether there was any exudation of inflammatory cells. The tissue sections were observed under a bright field microscope (DMi8, Leica, Wetzlar, German).

### 4.9. In Vivo Wound Healing in a Full-Thickness Skin Defect Model

Our animal experiment operation conforms to the requirements of the Animal Ethics Committee of Zhengzhou University. TM (Tegaderm) Film is a commercial wound dressing that can be used to protect skin wounds. Male Kunming mice weighing between 35 and 40 g were randomly divided into three groups, including the Control, TM Film, and HT_1_/QGA_0.3_ group. There was five mice in each group. A standard anesthesia procedure was performed on the mice, with an intraperitoneal injection of chloral hydrate (0.3 mg/kg body weight). The hair on the back of the mice was shaved, and a round wound with a diameter of 8 mm was created, which is the full-thickness skin injury model. After surgery, 100 µL of PBS was added to the wound in the control group, TM Film was attached to the wound surface in the TM Film group, and 100 µL hydrogel was injected onto the wound in the HT_1_/QGA_0.3_ group. The wound photographs were taken on the 1st, 3rd, 7th, and 14th day after treatment to compare the rate of wound contraction in each group.

### 4.10. Statistical Analysis

At least three independent replications for each data set of all the experiments were performed and the results are reported as mean ± SD. The statistical analysis was performed using GraphPad Prism via the Dunnett’s post-hoc test as part of one- or two-way ANOVA at the probabilities of * *p* < 0.05, ** *p* < 0.01, *** *p* < 0.001, and **** *p* < 0.0001 to show the levels of statistical significance.

## Data Availability

Data are available from the authors. Samples of the compounds are available from the authors.

## Supplementary Materials

The following are available online at https://www.mdpi.com/article/10.3390/gels7040204/s1, Figure S1: (a) 1H NMR spectra of HA and HT conjugate; (b) 1H NMR spectra of QCS and QGA conjugate.
