# Correction: Ren et al. Injectable and Antioxidative HT/QGA Hydrogel for Potential Application in Wound Healing. *Gels* 2021, *7*, 204

**DOI:** 10.3390/gels9090681

**Published:** 2023-08-24

**Authors:** Yikun Ren, Dan Zhang, Yuanmeng He, Rong Chang, Shen Guo, Shanshan Ma, Minghao Yao, Fangxia Guan

**Affiliations:** School of Life Science, Zhengzhou University, 100 Science Road, Zhengzhou 450001, China; renyikun1996@126.com (Y.R.); z13132572181@163.com (D.Z.); h1914375052@163.com (Y.H.); a15738343526@163.com (R.C.); 15137690960@163.com (S.G.); mashanshan84@163.com (S.M.)

## Error in Figure

In the original publication [1], there was a mistake in Figure 6 as published. In Figure 6b, the BMSCs live/dead staining photos have been misused in HT_1_/QGA_0.1_ and HT_1_/QGA_0.3_ groups for 24 h. Thus, we have replaced both pictures with our duplicated data. The corrected Figure 6 appears below. The authors state that the scientific conclusions are unaffected. This correction was approved by the Academic Editor. The original publication has also been updated.

## Figures and Tables

**Figure 6:**
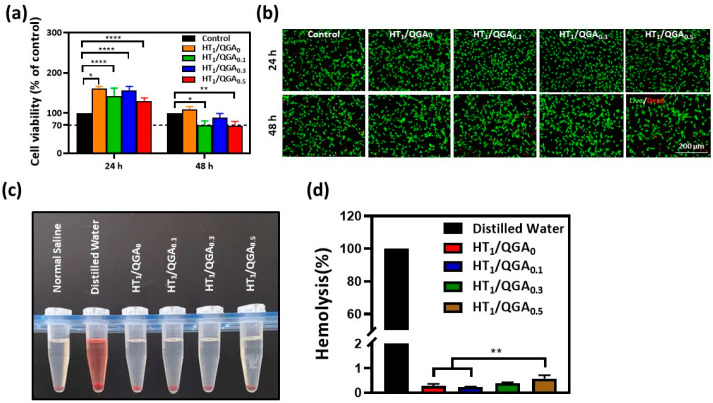
(**a**) BMSCs viability of HT/QGA hydrogel extracts by CCK-8; (**b**) live (Calcein-AM)/dead (PI) dyeing of BMSCs; (**c**) photograph of hemolysis test and (**d**) hemolysis ratio of HT/QGA hydrogels. * *p* < 0.05, ** *p* < 0.01, **** *p* < 0.0001, mean ± SD, *n* = 3.
