# Supplementary material for: Injectable and Antioxidative HT/QGA Hydrogel for Potential Application in Wound Healing
Source: Gels. 2021 Nov 9;7(4):204. doi: 10.3390/gels7040204 (PMC8628698; doi:10.3390/gels7040204)
Supplement: Supplementary file 1 [file gels-07-00204-s001.zip › gels-1450958-supplementary.pdf]

gels

MDPI

Article

Injectable and Antioxidative HT/QGA Hydrogel for Potential Application in Wound Healing

Yikun Ren †, Dan Zhang †, Yuanmeng He, Rong Chang, Shen Guo, Shanshan Ma, Minghao Yao * and Fangxia Guan *

The supplementary data

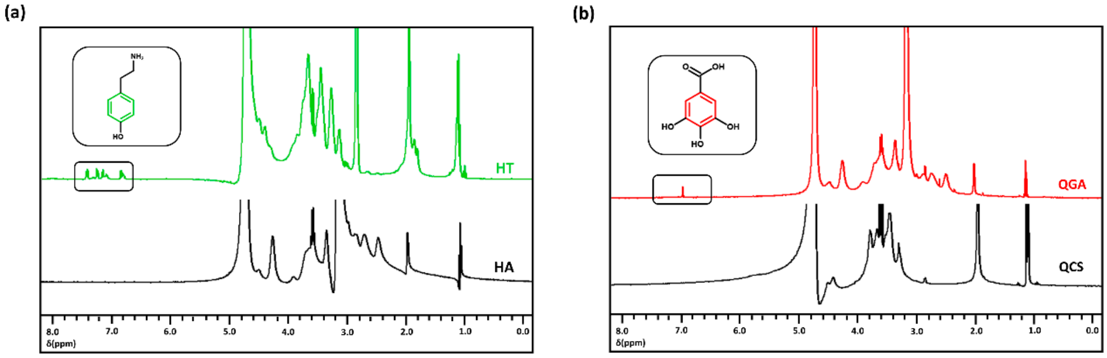

Figure S1. (a) ^{1}H NMR spectra of HA and HT conjugate; (b) ^{1}H NMR spectra of QCS and QGA conjugate.
